# Comorbidity Patterns and Management in Inpatients with Endocrine Diseases by Age Groups in South Korea: Nationwide Data

**DOI:** 10.3390/jpm14010042

**Published:** 2023-12-28

**Authors:** Sung-Soo Kim, Hun-Sung Kim

**Affiliations:** 1 Department of Healthcare Management, Cheongju University, Cheongju 28503, Republic of Korea; mra7033@naver.com; 2 Department of Medical Informatics, College of Medicine, The Catholic University of Korea, Seoul 06591, Republic of Korea; 3 Division of Endocrinology and Metabolism, Department of Internal Medicine, Seoul St. Mary’s Hospital, College of Medicine, The Catholic University of Korea, Seoul 06591, Republic of Korea

**Keywords:** endocrine diseases, comorbidity, diabetes mellitus, hypertension

## Abstract

This study aimed to examine comorbidity associations across age groups of inpatients with endocrine diseases as the primary diagnosis throughout the life cycle to develop an effective management strategy. Data were obtained from the Korean National Hospital Discharge In-depth Injury Survey (KNHDS) from 2006 to 2021, involving 68,515 discharged patients aged ≥ 19 years with a principal diagnosis of endocrine disease. A database was constructed for analysis, extracting general characteristics and comorbidities. Employing R version 4.2.3, the Chi-squared test and the Apriori algorithm of ARM (association rule mining) were used for analyzing general characteristics and comorbidity associations. There were more women (53.1%) than men (46.9%) (*p* < 0.001, with women (61.2 ± 17.2) having a higher average age than men (58.6 ± 58.6) (*p* < 0.001). Common comorbidities include unspecified diabetes mellitus; essential (primary) hypertension; unspecified diabetes mellitus; and other disorders of fluid, electrolyte, and acid-base balance. Notably, type 2 diabetes mellitus, disorders of lipoprotein metabolism and other lipidemia, polyneuropathy in diseases classified elsewhere, retinal disorders in diseases classified elsewhere, and essential (primary) hypertension prevail across all age groups. Association rules further highlight specific comorbidities appearing selectively in certain age groups. In conclusion, establishing a management strategy for comorbidities in patients with a primary diagnosis of an endocrine disorder is necessary.

## 1. Introduction

Endocrinology is a medical field that deals with various metabolic diseases caused by changes in hormones or various substances secreted in the body [1], such as the hypothalamus, pancreas, thyroid gland, parathyroid gland, and adrenal gland. Except for surgical cases, most endocrine diseases require lifelong medication and control [2]. Among them, diabetes mellitus (DM), hypertension, and dyslipidemia are representative metabolic diseases [3] that appear together in the form of various diseases rather than one disease because they increase with age [4].

When hospitalized in the endocrinology department, patients may require surgery in some cases; however, in most cases, the reason for admission is diagnosis, confirmation, and differential diagnosis of endocrine disease through hormone testing [5]. Its purpose is to maintain a normal hormonal state by controlling it with medications [6,7]. Taking DM as an example of a representative endocrine disease, the main purpose of hospitalization is blood glucose control, but screening tests for various diabetic complications are also performed to prevent the occurrence of early diabetic complications [8]. This is not only because comorbidities increase with age but also because diabetes itself causes chronic complications [8]. The American Diabetes Association (ADA) and Korean Diabetes Association (KDA) recommend assessing comorbidities before prescribing DM drugs [9,10]. Therefore, in patients hospitalized for endocrine diseases, including diabetes, various concomitant diseases must be managed simultaneously in addition to the main disease for which hospitalization is intended.

We predict that patients hospitalized for endocrine diseases will have differences in comorbidity patterns by age group. Identifying this provides the basis for effective patient management. This study aimed to classify the relationships between comorbidities by age and to lay the foundation for deriving efficient management strategies for concomitant diseases by screening for concomitant diseases in addition to the principal diagnosis of hospitalization.

## 2. Materials and Methods

### 2.1. Materials and Study Population

We used data from the Korea National Hospital Discharge In-Depth Injury Survey (KNHDIS). The KNHDIS is an annual sampling of the medical records of patients discharged from hospitals with more than 100 beds conducted by the Korea Disease Control and Prevention Agency [11]. The sampling method was two-stage stratified cluster sampling, with primary sampling of medical institutions as the sample frame and secondary sampling of patients in the sampled hospitals.

Data consisted of patient sociodemographic information, including sex and age; hospital visit information, including admission and discharge dates; diagnosis codes; surgery codes; and injury-related information. To conduct this study, we enrolled 3,678,866 discharged patients whose data were collected from the KNHDIS database between 2006 and 2021. Among adult males and females aged 19 years and older, 68,515 patients with a primary diagnosis of endocrine system diseases (E00–E90, International Classification of Diseases, 10th Revision [ICD–10]) were selected as the final study population.

### 2.2. Variables and Measures

The variables included patient characteristics, principal diagnoses, and other diagnoses. Patient characteristics included sex, age, insurance type, admission route, treatment outcome, resource days, death, surgery, comorbidities, and hospital bed size. Age was categorized into 19–44, 45–64, 65–74, and ≥75 years using the life cycle characteristics [12]. The insurance type was categorized into health insurance, medicaid type 1, medicaid type 2, and others according to the Korean National Health Insurance System [13]. Hospitalization was classified as outpatient, emergency, or other. Treatment outcomes were categorized as improved, unimproved, death, or other. The length of stay was calculated as the number of days between the date of admission and the date of discharge using the single-entry method. Surgery codes were used to classify whether surgery was performed, and comorbidities were classified by checking whether comorbidities were diagnosed. Hospital size was categorized into 100–200, 300–499, 500–999, and ≥1000 beds. Primary diagnoses and comorbidities are standardized ICD–10 (International Classification of Diseases, 10th Revision) code data. Disease groups for the association analysis were classified by a three-digit intermediate classification.

### 2.3. Data Analysis

We used MySQL software (Version 8.0) to efficiently manage over 3 million cases of research data. MySQL is a database management tool widely used for data management in the healthcare field [14]. The final extracted data were analyzed using R version 4.2.3. The general characteristics of the study participants according to sex were compared using the Chi-squared test. The distribution of comorbidities was graphically visualized using the “itemFrequencyPlot” command. Finally, the Apriori algorithm for ARM (association rule mining) was used to identify the association between comorbidities. The Apriori algorithm is widely used to analyze frequent item sets and association rules, as well as in the fields of finance, marketing, and healthcare [15,16,17,18,19].

The key metrics for identifying the association rules were support, confidence, and lift [20]. Support refers to the frequency at which a particular item occurs in the entire dataset. In this study, support (A → B) refers to the proportion of all participants with diseases A and B. Confidence is the frequency of items A and B occurring simultaneously with item A. In this study, confidence (A → B) means the proportion of patients with disease A who have both disease A and disease B. Lift is a metric used to determine how effective an association rule is in practice. This is the ratio of the probability of two items occurring simultaneously to the probability that they occur independently. In this study, lift (A → B) is the ratio of the probability of disease A and disease B occurring together to the probability of disease A and disease B occurring independently. If the lift value is 2, the probability of the two diseases occurring together is twice as high as if they occurred independently. Therefore, a rule must have a lift value of at least 1 to be meaningful. Lift is used to measure the strength of association rules; however, this study analyzed interest support (IS), which considers both support and lift. IS (A → B) is the square root of the product of support (A → B) and lift (A → B). The formula is as follows:

(1) $$Support\;\left(A\to B\right)=\frac{Number\;of\;patients\;with\;disease\;A\;and\;disease\;B}{Total\;number\;of\;patients}$$

(2) $$Confidence\;\left(A\to B\right)=\frac{Number\;of\;patients\;with\;disease\;A\;and\;disease\;B}{Number\;of\;patients\;with\;disease\;A}$$

(3) $$Lift\;\left(A\to B\right)=\frac{P\left(disease\;A,\;disease\;B\right)}{P\left(disease\;A\right)\cdot P\left(disease\;B\right)}$$

(4) $$IS\;\left(A\to B\right)=\sqrt{Support\;\left(A\to B\right)\;\times \;Lift\;\left(A\to B\right)}$$

To extract useful association rules, minimum criteria for support and reliability were iteratively fitted. If the minimum criteria are too large, no useful association rules can be found; if they are too low, many unhelpful association rules occur. Considering the characteristics of previous studies and the data of this study, final support > 0.02 and reliability > 0.1 were applied. To check the pattern of comorbidities by age group, we visualized each as a network graph using the arulesViz library in R. The arulesViz helps users explore and understand rule-based models [21,22]. Finally, the major comorbidities shown in the network graph by age group are summarized in for management.

## 3. Results

### 3.1. Characteristics of Study Subjects and Distribution of Comorbidities

**Figure 1:**
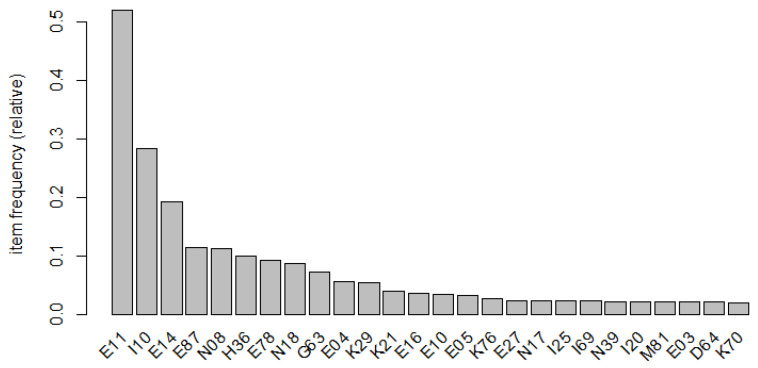
Plot of comorbidity frequency. D64, Other anemias; E03, Other hypothyroidism; E04, Other nontoxic goiter; E05, Thyrotoxicosis [hyperthyroidism]; E10, Type 1 diabetes mellitus; E11, Type 2 diabetes mellitus; E14, Unspecified diabetes mellitus; E16, Other disorders of pancreatic internal secretion; E27, Other disorders of adrenal gland; E78, Disorders of lipoprotein metabolism and other lipidemias; E87, Other disorders of fluid, electrolyte, and acid-base balance; G63, Polyneuropathy in diseases classified elsewhere; H36, Retinal disorders in diseases classified elsewhere; I10, Essential (primary) hypertension; I20, Angina pectoris; I25, Chronic ischemic heart disease; I69, Sequelae of cerebrovascular disease; K21, Gastro-esophageal reflux disease; K29, Gastritis and duodenitis; K70, Alcoholic liver disease; K76, Other diseases of liver; M81, Osteoporosis without pathological fracture; N08, Glomerular disorders in diseases classified elsewhere; N17, Acute renal failure; N18, Chronic kidney disease; N39, Other disorders of urinary system.

The characteristics of the participants according to sex are shown in Table 1. Of the 68,515 study participants, 46.9% were male and 53.1% were female. The average age of women was 61.2 years, compared to 58.6 years for men. By age group, 45–64-year-olds were the most represented, with 45.2% men and 32.5% women. Men were least represented in the 75+ age group (16.0%), while women were least represented in the 19–44 age group (18.1%). The insurance type was national health for more than 80% of men and women. The admission route was 70% outpatient for both men and women, and the rest were admitted through an emergency department; therefore, there was no significant difference in the distribution by sex. The treatment outcome was better than 95%, but 3.4% of males and 2.8% of females did not improve. The mortality rate was 1.4% in men and 1.0% in women—slightly higher in men. The length of stay was 12.0 days for men, and 9.6 days for women—2.4 days longer for men. Overall, 17.2% of men and 17.9% of women underwent surgery during their stay. A total of 82.5% of men had comorbidities, while 80.2 percent of women were diagnosed with comorbidities. In terms of hospital bed size, 42.9% of men and 44.8% of women were admitted to hospitals with 500–999 beds. Next, 29.2% of men and 27.0% of women were admitted to hospitals with 100–299 beds. The frequency of comorbidities among the study participants is shown in Figure 1. Type 2 is the most common type of diabetes mellitus. This was followed by essential (primary) hypertension, unspecified diabetes mellitus, lipoprotein metabolism disorders, other lipidemias, and glomerular disorders in diseases classified elsewhere.

### 3.2. Overall Association Rule Mining

**Figure 2:**
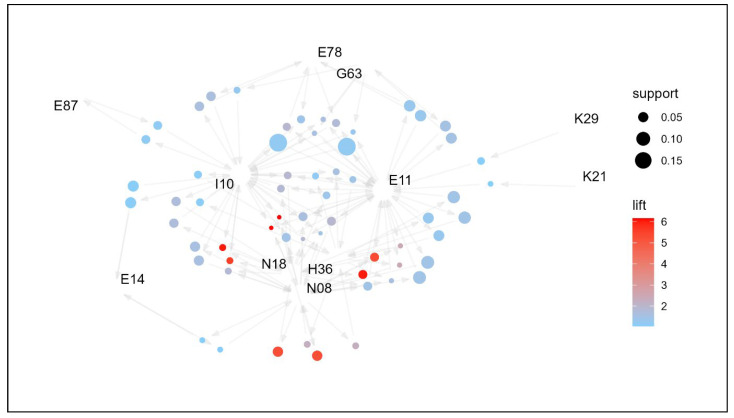
Network graph of the association of comorbidities in patients whose principal diagnosis is endocrine, nutritional, and metabolic diseases (age: >19 years).

The results of the comorbidity association analysis of the study participants are shown in Table 2. There were 61 association rules with a lift greater than one, and they were sorted in descending order by IS, which is an indicator of the strength of the association considering support and lift. The most relevant rule was “N08 → N18” (lift = 5.239, IS = 0.519). This means that “glomerular disorders in diseases classified elsewhere” and “chronic kidney disease” were 5.239 times more likely to occur together than independently. The reverse path “N18 → N08” shows the same association. The second highest association rule is “E11, N18 → N08” (lift = 6.017, IS = 0.485). This has a higher lift than the first association rule but a slightly lower support of 0.039; thus, the association strength metric IS is linked to the second. This means that “type 2 diabetes mellitus, chronic kidney disease”, and “glomerular disorders in diseases classified elsewhere” were 6.017 times more likely to occur together than independently. Next, the association rule “I10 → E11” between “essential (primary) hypertension” and “type 2 diabetes mellitus” had the third highest strength of association (lift = 1.217, IS = 0.467). Patients with endocrine disorders are 1.217 times more likely to have “essential (primary) hypertension” and “type 2 diabetes mellitus” together than independently. With a support value of 0.179, the number of patients with these two diseases together was very high (17.9%), making it the third highest lift rule, although its lift value was lower than those of the first two association rules. Subsequent association rules can be interpreted in the same manner. Of the 61 association rules involved with type 2 diabetes mellitus (E11) and essential (primary) hypertension (I10), two of the other association rules were described earlier, and the remaining two are association rules 32 and 33, “H36 → N08” and “N08 → H36” (lift = 2.275, IS = 0.243). This means that “glomerular disorders in diseases classified elsewhere” and “retinal disorders in diseases classified elsewhere” are 2.275 times more likely to be diagnosed together than independently. Overall, 63.9% of the 61 association rules included type 2 diabetes mellitus (E11), 60.7% included essential (primary) hypertension (I10), and 34.4% included both. The association rules for all subjects aged 19 years are visualized in a network graph, as shown in Figure 2. Type 2 diabetes mellitus (E11) and essential (primary) hypertension (I10) showed the most common associations, with the remaining conditions forming an association pathway.

### 3.3. Comorbidities Association Rule Mining by Age Group

**Figure 3:**
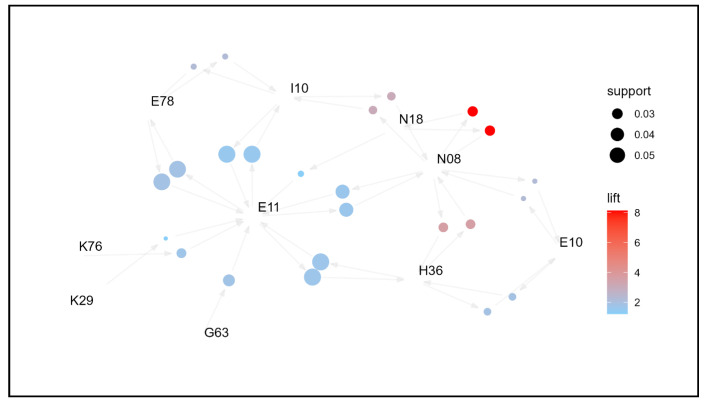
Network graph of the association of comorbidities in patients whose principal diagnosis is endocrine, nutritional, and metabolic diseases (age: 19–44 years old).

**Figure 4:**
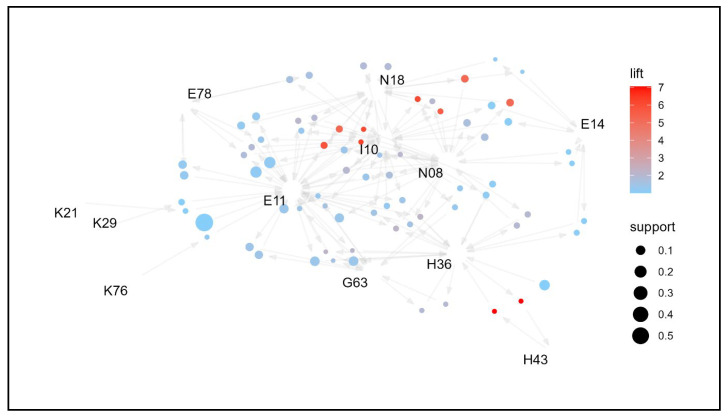
Network graph of the association of comorbidities in patients whose principal diagnosis is endocrine, nutritional, and metabolic diseases (age: 45–64 years old).

**Figure 5:**
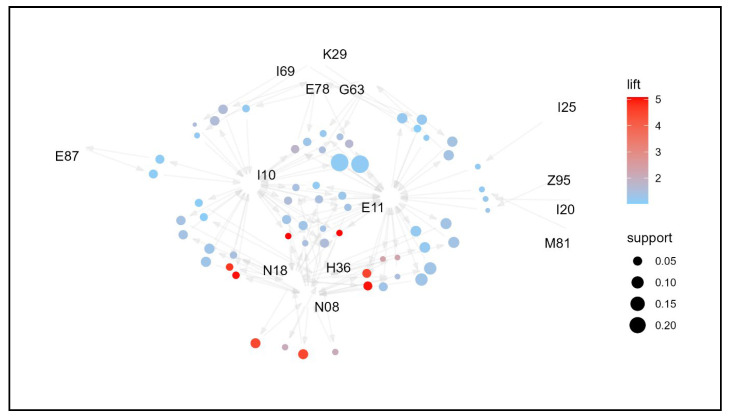
Network graph of the association of comorbidities in patients whose principal diagnosis is endocrine, nutritional, and metabolic diseases (age: 65–74 years old).

**Figure 6:**
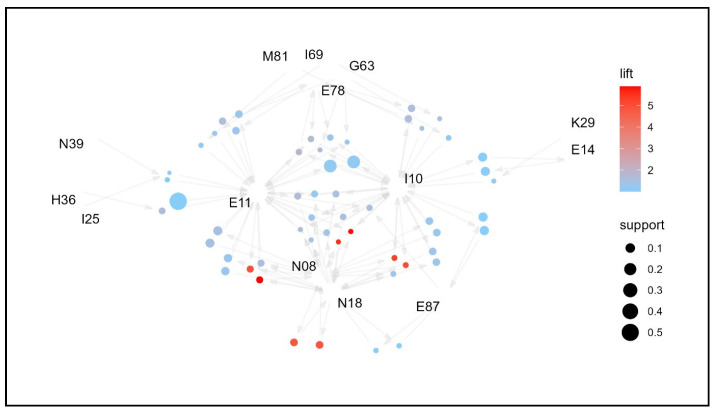
Network graph of the association of comorbidities in patients whose principal diagnosis is endocrine, nutritional, and metabolic diseases (age: >75 years old).

To identify the association rules of comorbidities by age group and examine management strategies, network graphs were created for each age group (Figure 3, Figure 4, Figure 5 and Figure 6). Based on the network graph, we summarized the distribution of major comorbidities (Table 3). Overall, type 2 diabetes mellitus (E11), disorders of lipoprotein metabolism and other lipidemias (E78), polyneuropathy in diseases classified elsewhere (G63), retinal disorders in diseases classified elsewhere (H36), essential (primary) hypertension (I10), gastritis and duodenitis (K29), glomerular disorders in diseases classified elsewhere (N08), and chronic kidney disease (N18) were association rules in all age groups. However, other disorders of fluid, electrolyte, and acid-base balance (E87), sequelae of cerebrovascular disease (I69), and osteoporosis without pathological fracture (M81) were only present in the ≥65 years age group, and chronic ischemic heart disease (I25) and other disorders of the urinary system (N39) formed association pathways only in the 75+ age group. In contrast, type 1 diabetes mellitus (E10) was only observed in the 19–44 years age group, gastro-esophageal reflux disease (K21) in the 45–64 age group, and other liver diseases (K76) in the <65 years age group. Unspecified diabetes mellitus (E14) was intermittently distributed among 45–64-year-olds and ≥75-year-olds.

## 4. Discussion

Except for some endocrine diseases that require confirmation/differentiation of specific diseases, most endocrine diseases are controlled with medication in outpatient settings rather than hospitalization [2]. Hospitalization for endocrine diseases involves the prompt treatment of patients with severe hormonal imbalance or hyperglycemia [23]. In most cases, endocrine diseases occur together with other diseases as people age [4]. Therefore, most patients admitted to the endocrinology department often have other diseases, and the management of these concomitant diseases has a profound impact on the treatment effect, course, and follow-up of the disease, which is the reason for hospitalization.

DM is the most common disease among hospitalized endocrinology patients [3]. When a patient with DM is admitted to the endocrinology department, it is used for blood glucose control because the blood glucose level is either very high or very low. In these cases, the main purpose is to control blood glucose levels during hospitalization. If a patient with DM is hospitalized to control blood glucose levels, a DM complication test is also performed. Retinopathy, nephropathy, and neuropathy are well known as the three major complications associated with DM [24]. These three well-known complications are consistent with the results of this study. In fact, the coexistence rate between DM and retinopathy was 1.46 times, kidney disease 1.277–1.451 times, and neuropathy 1.522 times, which was very high compared to other diseases. In addition, the possibility of accompanying hypertension or dyslipidemia was high.

DM is already well known as the most common cause of CKD [25]. In this study, CKD was the most common comorbidity in patients compared to other diseases. Although this is already well known and clinically considered, the results of this study are consistent with actual clinical results. The relative risk of developing CKD in patients with DM is approximately 1.3–4.6 times higher [26,27]. In addition, DM accounts for the largest proportion of diseases requiring dialysis therapy [25]. Therefore, medical staff should focus on the early diagnosis of nephropathy, which is frequently accompanied by DM. Early detection and treatment of diabetic nephropathy are known to reduce the rate of disease worsening in CKD [28].

Diabetic retinopathy is a complication in approximately 25% of DM cases [29,30]. DM and retinopathy had a high coexistence rate in this study. In DM, the active control of blood glucose levels and blood pressure can prevent or delay the development of retinopathy [31,32]. Patients with DM usually undergo annual examinations if retinopathy is not found during the initial ophthalmological examination [33]. However, considering cost-effectiveness, it is controversial whether the examination cycle can be extended to low-risk groups of diabetic retinopathies [34]. However, based on the results of this study, it is better to proceed more actively than extend the screening cycle for diabetic retinopathy [35].

This study also found a high incidence of diabetic neuropathy. The prevalence of diabetic neuropathy in Korea is widely known to be 25–53% [36,37,38]. Diabetic neuropathy also appears relatively early and has a high incidence, but it is easily ignored, and its detection is often delayed. This is because a diagnosis is possible only after ruling out neuropathy caused by other diseases [39]. Early diagnosis and treatment are important to improve symptoms, reduce hospitalization mortality, reduce sequelae, and improve the quality of life [40]. Therefore, more attention should be paid to diabetic neuropathy, which is likely to be a comorbidity of DM.

This study emphasizes that nephropathy, retinopathy, neuropathy, and hypertension are all interrelated owing to the nature of chronic endocrine diseases. Those with DM and hypertension were 1.451 and 1.581 times more likely to develop CKD, respectively. However, patients with diabetes and high blood pressure were 1.928 times more likely to have CKD than kidney disease. Other diseases also showed the same trend, showing that comorbidities were higher in patients with two diseases than in those with one disease. Ultimately, most chronic endocrinological diseases are interconnected, and it is important to manage the overall comprehensive disease rather than managing only one disease.

Rather than these known comorbidities, we needed to analyze relatively lesser-known comorbidities. In this study, electrolyte and acid-base imbalances were present in many cases of hypertension. Various clinical inferences can be drawn from this issue. For example, hyperkalemia that occurs when taking blood pressure medications ACEI/AEB [41], electrolyte imbalance caused by CKD [42], and, less commonly, hypertension and hypokalemia caused by aldosteronism [43] can be inferred. This study focused on elderly people aged over 65 years. In many elderly people, DM or hypertension has already progressed significantly; therefore, it can be inferred that electrolyte imbalance occurs owing to various causes [44]. Importantly, although we do not know the cause of electrolyte and acid-base imbalances, it is recommended to periodically measure electrolyte and acid-base levels in patients hospitalized in the endocrinology department and in those over 65 years of age with hypertension. This appears reasonable. What we would like to emphasize in this study is the need for active testing and follow-up for specific diseases and accompanying diseases, although larger-scale prospective studies are needed.

In this study, GERD, which is concentrated in patients with DM aged 45–64 years, and gastritis and duodenitis, which occur in all age groups, were also noteworthy. Damage to autonomic nerve cells reduces gastrointestinal motor function [45]. Diabetic gastroparesis causes symptoms of gastroparesis and affects nutritional intake in patients with diabetes; therefore, it can have a significant adverse effect on blood glucose level control during hospitalization due to repeated hyperglycemia and hypoglycemia. However, clinically, it is less well known than the other complications mentioned above; therefore, patients’ symptoms can be easily overlooked. However, according to the results of this study, DM exists as a high comorbidity, and doctors should accurately recognize this situation and approach patients accordingly.

The greatest advantage of this study is that it used national big data accumulated over a period of more than 10 years. The second advantage is that it targeted hospitalized patients. Hospitalized patients are more severely ill and have many comorbidities. Therefore, inpatients are much more important than outpatients in terms of early identification and management of comorbidities. It would be more effective if early management is initiated by screening, diagnosing, and differentiating comorbidities in patients with severe endocrine diseases. Testing is easier for hospitalized patients than for outpatients, and immediate feedback based on the test results is possible. Based on the results of this study, hospitalized patients will have a good prognosis if additional diseases are found after additional tests for concomitant diseases and appropriate responses are obtained.

This study has several limitations due to its retrospective cohort design [46,47]. First, the causal relationship between the two diseases cannot be identified; only the accompanying correlation can be identified because various confounding variables can affect it. However, as this study was conducted in patients whose principal diagnosis was endocrine disease, it is expected that various clinicians will apply it in clinical practice for various reasons and discussions. Additional large-scale prospective studies are needed to confirm these findings. Second, because most patients admitted to the endocrinology department had DM, most of the comorbidities were associated with DM, and relatively few other endocrinological diseases were included. Finally, when differentiating diseases using ICD–10 diagnostic codes, there were cases in which similar types of diseases were grouped together (e.g., ICD–10 N08 and N18). However, because this case is subject to interpretation by clinicians, it does not have a significant impact on the conclusions of the study.

## 5. Conclusions

Endocrine diseases, including DM, are complex conditions accompanied by multiple diseases, rather than a single disease. Understanding the characteristics of these endocrinological diseases is crucial for effective patient management. Network graphs are useful for visually representing interrelationships and connections between comorbidities. Lifelong treatment and comprehensive disease management prove pivotal in reducing complications and enhancing the quality of life. This serves as the ultimate objective for medical professionals managing patients admitted to the endocrinology department. While conducting comprehensive tests is routine for these patients, acknowledging age-specific comorbidity patterns and tailoring treatments accordingly is advisable. Future approaches must consider individual patient characteristics, various test results, and prescribed medications for personalized comorbidity assessments, requiring comprehensive lifestyle management and individualized treatment strategies.

## Figures and Tables

**Table 1:** Baseline characteristics of study population.

Variables	Male	Female	*p*
	N (%) or Mean ± SD	N (%) or Mean ± SD	
N	32,140	36,375	
Age (year)	58.6 ± 15.0	61.2 ± 17.2	<0.001
Age group			<0.001
19–44	5653 (17.6%)	6575 (18.1%)	
45–64	14,523 (45.2%)	11,813 (32.5%)	
65–74	6815 (21.2%)	8552 (23.5%)	
≥75	5149 (16.0%)	9435 (25.9%)	
Insurance type			<0.001
National health	26,216 (81.6%)	30,728 (84.5%)	
Medicaid type 1	4759 (14.8%)	4803 (13.2%)	
Medicaid type 2	663 (2.1%)	600 (1.6%)	
Others	502 (1.6%)	244 (0.7%)	
Admission route			0.862
Emergency	9799 (30.5%)	11,048 (30.4%)	
Outpatient	22,310 (69.4%)	25,288 (69.5%)	
Others	31 (0.1%)	39 (0.1%)	
Treatment outcome			<0.001
Improved	30,566 (95.1%)	34,958 (96.1%)	
Not improved	1085 (3.4%)	1032 (2.8%)	
Death	453 (1.4%)	350 (1.0%)	
Others	36 (0.1%)	35 (0.1%)	
Length of stay (day)	12.0 ± 19.0	9.6 ± 16.5	<0.001
Death Y/N			<0.001
Yes	453 (1.4%)	350 (1.0%)	
No	31,687 (98.6%)	36,025 (99.0%)	
Operation Y/N			0.010
Yes	5520 (17.2%)	6521 (17.9%)	
No	26,620 (82.8%)	29,854 (82.1%)	
Comorbidity Y/N			<0.001
Yes	26,522 (82.5%)	29,162 (80.2%)	
No	5618 (17.5%)	7213 (19.8%)	
Bed size			<0.001
100–299	9400 (29.2%)	9812 (27.0%)	
300–499	4453 (13.9%)	5153 (14.2%)	
500–999	13,784 (42.9%)	16,306 (44.8%)	
≥1000	4503 (14.0%)	5104 (14.0%)	

Abbreviations: SD, standard deviation.

**Table 2:** Association rule mining between comorbidity of the study population.

No	Rules	N	Support	Confidence	Lift	IS
1	N08 → N18	3523	0.051	0.457	5.239	0.519
2	N18 → N08	3523	0.051	0.589	5.239	0.519
3	E11, N18 → N08	2683	0.039	0.677	6.017	0.485
4	I10 → E11	12,259	0.179	0.632	1.217	0.467
5	E11 → I10	12,259	0.179	0.344	1.217	0.467
6	E11, N08 → N18	2683	0.039	0.462	5.294	0.455
7	I10, N08 → N18	1811	0.026	0.525	6.018	0.399
8	I10, N18 → N08	1811	0.026	0.626	5.561	0.383
9	E11, I10, N08 → N18	1429	0.021	0.537	6.159	0.358
10	E11, I10, N18 → N08	1429	0.021	0.689	6.128	0.358
11	E11 → N08	5808	0.085	0.163	1.451	0.351
12	N08 → E11	5808	0.085	0.754	1.451	0.351
13	H36 → E11	5256	0.077	0.757	1.457	0.334
14	E11 → H36	5256	0.077	0.148	1.457	0.334
15	G63 → E11	3973	0.058	0.791	1.522	0.297
16	E11 → G63	3973	0.058	0.112	1.522	0.297
17	E78 → E11	4406	0.064	0.683	1.314	0.291
18	E11 → E78	4406	0.064	0.124	1.314	0.291
19	N08 → I10	3449	0.050	0.448	1.581	0.282
20	I10 → N08	3449	0.050	0.178	1.581	0.282
21	E11, I10 → N08	2659	0.039	0.217	1.928	0.274
22	N18 → E11	3964	0.058	0.663	1.277	0.272
23	E11 → N18	3964	0.058	0.111	1.277	0.272
24	E78 → I10	3008	0.044	0.466	1.647	0.269
25	I10 → E78	3008	0.044	0.155	1.647	0.269
26	I10 → N18	2895	0.042	0.149	1.711	0.269
27	N18 → I10	2895	0.042	0.484	1.711	0.269
28	E14 → I10	4079	0.060	0.309	1.093	0.255
29	I10 → E14	4079	0.060	0.210	1.093	0.255
30	E11, N08 → I10	2659	0.039	0.458	1.617	0.251
31	E11, I10 → E78	2226	0.032	0.182	1.928	0.250
32	H36 → N08	1778	0.026	0.256	2.275	0.243
33	N08 → H36	1778	0.026	0.231	2.275	0.243
34	E11, I10 → N18	2073	0.030	0.169	1.938	0.242
35	E11, E78 → I10	2226	0.032	0.505	1.785	0.241
36	I10, N08 → E11	2659	0.039	0.771	1.484	0.240
37	N08, N18 → E11	2683	0.039	0.762	1.466	0.240
38	E11, N18 → I10	2073	0.030	0.523	1.847	0.236
39	E11, N08 → H36	1467	0.021	0.253	2.491	0.231
40	E11, H36 → N08	1467	0.021	0.279	2.481	0.230
41	N08, N18 → I10	1811	0.026	0.514	1.816	0.219
42	E78, I10 → E11	2226	0.032	0.740	1.425	0.215
43	E87 → I10	2614	0.038	0.333	1.178	0.212
44	I10 → E87	2614	0.038	0.135	1.178	0.212
45	I10, N18 → E11	2073	0.030	0.716	1.379	0.204
46	H36, I10 → E11	1789	0.026	0.825	1.589	0.204
47	E11, N08, N18 → I10	1429	0.021	0.533	1.882	0.198
48	E11, I10 → H36	1789	0.026	0.146	1.439	0.194
49	E11, I10 → G63	1483	0.022	0.121	1.650	0.189
50	H36 → I10	2168	0.032	0.312	1.102	0.187
51	I10 → H36	2168	0.032	0.112	1.102	0.187
52	G63, I10 → E11	1483	0.022	0.829	1.596	0.186
53	H36, N08 → E11	1467	0.021	0.825	1.588	0.184
54	G63 → I10	1789	0.026	0.356	1.258	0.181
55	I10, N08, N18 → E11	1429	0.021	0.789	1.519	0.178
56	K29 → E11	2052	0.030	0.549	1.056	0.178
57	E11, H36 → I10	1789	0.026	0.340	1.202	0.177
58	E11, G63 → I10	1483	0.022	0.373	1.319	0.169
59	N08 → E14	1560	0.023	0.202	1.052	0.155
60	E14 → N08	1560	0.023	0.118	1.052	0.155
61	K21 → E11	1533	0.022	0.553	1.064	0.154

Abbreviations: IS, Interest Support; E11, Type 2 diabetes mellitus; E14, Unspecified diabetes mellitus; E78, Disorders of lipoprotein metabolism and other lipidemias; E87, Other disorders of fluid, electrolyte, and acid-base balance; G63, Polyneuropathy in diseases classified elsewhere; H36, Retinal disorders in diseases classified elsewhere; I10, Essential (primary) hypertension; K21, Gastro-esophageal reflux disease; K29, Gastritis and duodenitis; N08, Glomerular disorders in diseases classified elsewhere; N18, Chronic kidney disease.

**Table 3:** Distribution of major comorbidities by age group because of association rule mining.

ICD-10	Code Description	Age Group				
		19~44	45~64	65~74	75+	All Age
E10	Type 1 diabetes mellitus	O				
E11	Type 2 diabetes mellitus	O	O	O	O	O
E14	Unspecified diabetes mellitus		O		O	O
E78	Disorders of lipoprotein metabolism and other lipidemias	O	O	O	O	O
E87	Other disorders of fluid, electrolyte, and acid-base balance			O	O	O
G63	Polyneuropathy in diseases classified elsewhere	O	O	O	O	O
H36	Retinal disorders in diseases classified elsewhere	O	O	O	O	O
H43	Disorders of vitreous body		O			
I10	Essential (primary) hypertension	O	O	O	O	O
I20	Angina pectoris			O		
I25	Chronic ischemic heart disease			O	O	
I48	Atrial fibrillation and flutter					
I69	Sequelae of cerebrovascular disease			O	O	
K21	Gastro-esophageal reflux disease		O			O
K29	Gastritis and duodenitis	O	O	O	O	O
K76	Other diseases of liver	O	O			
M81	Osteoporosis without pathological fracture			O	O	
N08	Glomerular disorders in diseases classified elsewhere	O	O	O	O	O
N17	Acute renal failure					
N18	Chronic kidney disease	O	O	O	O	O
N39	Other disorders of urinary system				O	
Z95	Presence of cardiac and vascular implants and grafts			O		

## Data Availability

Restrictions apply to the availability of these data. Data were obtained from KCDC and are available from https://www.cdc.go.kr/contents.es?mid=a20303010502 (accessed on 17 May 2023).
